# In Nonagenarians, Acute Kidney Injury Predicts In-Hospital Mortality, while Heart Failure Predicts Hospital Length of Stay

**DOI:** 10.1371/journal.pone.0077929

**Published:** 2013-11-06

**Authors:** Chia-Ter Chao, Yu-Feng Lin, Hung-Bin Tsai, Nin-Chieh Hsu, Chia-Lin Tseng, Wen-Je Ko, The HINT Study Group

**Affiliations:** 1 Nephrology division, Department of Internal Medicine, National Taiwan University Hospital, Taipei, Taiwan; 2 Department of Traumatology, National Taiwan University Hospital, Taipei, Taiwan; 3 Hospitalist in National Taiwan University Hospital, Taipei, Taiwan; S.G.Battista Hospital, Italy

## Abstract

**Background/Aims:**

The elderly constitute an increasing proportion of admitted patients worldwide. We investigate the determinants of hospital length of stay and outcomes in patients aged 90 years and older.

**Methods:**

We retrospectively analyzed all admitted patients aged >90 years from the general medical wards in a tertiary referral medical center between August 31, 2009 and August 31, 2012. Patients’ clinical characteristics, admission diagnosis, concomitant illnesses at admission, and discharge diagnosis were collected. Each patient was followed until discharge or death. Multivariate logistic regression analysis was utilized to study factors associated with longer hospital length of stay (>7 days) and in-hospital mortality.

**Results:**

A total of 283 nonagenarian in-patients were recruited, with 118 (41.7%) hospitalized longer than one week. Nonagenarians admitted with pneumonia (p = 0.04) and those with lower Barthel Index (p = 0.012) were more likely to be hospitalized longer than one week. Multivariate logistic regression analysis revealed that patients with lower Barthel Index (odds ratio [OR] 0.98; p = 0.021) and those with heart failure (OR 3.05; p = 0.046) had hospital stays >7 days, while patients with lower Barthel Index (OR 0.93; p = 0.005), main admission nephrologic diagnosis (OR 4.83; p = 0.016) or acute kidney injury (OR 30.7; p = 0.007) had higher in-hospital mortality.

**Conclusion:**

In nonagenarians, presence of heart failure at admission was associated with longer hospital length of stay, while acute kidney injury at admission predicted higher hospitalization mortality. Poorer functional status was associated with both prolonged admission and higher in-hospital mortality.

## Introduction

In the past century, we have witnessed the phenomenon of increasing life expectancy globally, as well as population aging [1]. This leads to an ever-increasing proportion of very old patients being admitted into hospitals [2], [3]. It is then imperative for clinicians to be familiar with the clinical features of the elderly population. Amongst these, the oldest-old patients (those aged ≥90 years), the nonagenarians and centenarians, appear to be a unique subgroup, with few articles specifically addressing this population [4].

Although aging itself is associated with organ function degeneration and susceptibility to various insults, older-old patients (aged >75 years) display distinct clinical characteristics from their younger-old (aged 65–75 years) counterparts, possibly due to survivor bias or to other undiscovered reasons [5]. Many conventional survival determinants do not apply in these older-old patients [5]–[7]. A large cohort study in Denmark revealed that such parameters as sociodemographic factors (marital status, educational level), smoking and alcohol consumption do not predict mortality in nonagenarians [7]. Another study in Spain likewise found that age and cardiovascular comorbidities were not associated with long-term survival in nonagenarians [8]. In addition, previous studies have been mostly community-based and investigate long-term prognosis instead of in-hospital mortality [4], [7], [9], [10]. Furthermore, none of these studies focus on the factors affecting hospital length of stay in nonagenarian in-patients.

We hypothesized that other factors, in addition to demographic profiles (age, gender) and also traditional vascular risk factors (diabetes mellitus [DM], coronary artery disease [CAD]) are predictive of nonagenarian hospital mortality and length of stay. We utilized a cohort of nonagenarians admitted to the general medical care units to assess these potential associated factors.

## Materials and Methods

### Ethical Consideration

This study was approved by the local institutional review board (IRB) (NO. 201112161RIC) of National Taiwan University Hospital (NTUH), Taipei, Taiwan. Since all identifiable information for all individuals in the study is encrypted to protect patient privacy, the local IRB waived the need to obtain patient consent (including written or oral form) for the current study.

### Study Design and Data Sources

In the current study, participants were identified and enrolled from acute general medical wards (AGMWs) in a tertiary medical center in Northern Taiwan. NTUH is a national referral center, with patient admitted from all over Taiwan. The admission criteria for AGMWs include all patients, ranked from 1 to 4 triage categories (of a total of 5 categories, with decreasing number denoting more severe illnesses and more unstable vital signs), with medical diagnoses (of any subspecialty) and requiring hospitalization for subsequent care, in the emergency department [11]. Surgical patients are not admitted to AGMW and thus were not enrolled. These AGMWs are run by hospitalists, a specialty associated with shorter hospital stays, higher efficiency and lower in-patient healthcare costs [11]. Subspecialty wards were not selected due to the lack of generalizability and the different admission criteria, while AGMW patients are more representative of the average in-patient population.

Patients were eligible if they were aged ≥90 years and admitted to an AGMW between August 31, 2009 and August 31, 2012. We recorded demographic data and comorbidities, as well as main and concomitant diagnoses at patient admission. Functional status (assessed by Barthel Index) was also documented at admission and discharge, except for those who died during hospitalization; for these patients, the Barthel Index used was the last one recorded. Barthel Index is a scale utilized to assess the performance status of patients in their activities of daily living (ADL) [12]. The 10 items within the scale include eating, bathing, grooming, dressing, defecation, urination, going to the toilet, getting out of bed, walking and stair-climbing. Each performance item is rated by points, with the sum of all items indicating the patient’s ADL. In our AGMWs, Barthel Index is routinely assessed by two independent case managers every 3–4 days, using a standardized format (Chinese version), with low inter-rater variability. The Charlson index was also recorded as a summary of each patient’s comorbidities [13]. Treatment variables, including receipt of cardiopulmonary resuscitation (CPR) and Do-Not-Resuscitate (DNR) code, were recorded. At discharge or death, we re-examined each patient’s discharge diagnoses and other complications during hospitalization.

### Variables, Endpoints and Outcomes

The definitions of all comorbidities were based on past medical history and compatible objective findings, as listed in Table 1. Concomitant illnesses at admission and discharge were also identified. Pneumonia was diagnosed if patients presented with a productive cough, fever and compatible radiologic findings. Acute pancreatitis was defined according to abdominal computed tomography (CT) findings and a more than 2-fold elevation of serum lipase levels. Ileus was defined according to clinical symptoms and compatible radiologic findings. Urinary tract infection (UTI) was diagnosed according to a positive urine culture of >10^5^ colony-forming units/ml. Acute kidney injury (AKI) was defined as serum creatinine levels >44.3 µmol/L (0.5 mg/dL) at admission compared with the patient’s pre-admission levels, according to the Risk-Injury-Failure-Loss-End stage (RIFLE) classification, a consensus proposed by the Acute Dialysis Quality Initiative (ADQI) group in 2004 [14]. This definition has been utilized widely to define, classify and predict the outcomes of AKI of different etiologies, with high accuracy and predictability. Ischemic stroke was defined by new-onset neurologic deficits with compatible neuroimaging results.

**Table 1:** Definitions of comorbidities used in the current study.

Comorbidities	Definition
DM	Diet-controlled status, or past insulin or oral hypoglycemic agents users more than one month, or present users
Hypertension	Previous anti-hypertensive agents users for more than one month, or present users, or blood pressure higher than 140/90 mmHg for 3 days after admission
CAD	History of compatible coronary angiographic findings
HF	History of compatible symptoms with echocardiography findings
PAOD	History of compatible vascular duplex or angiographic findings
PUD	History of compatible panendoscopic findings
COPD	As diagnosed by a certified pulmonologist in the past and receiving relevant medications for more than one month, or present user
Dementia	As diagnosed by a certified neurologist in the past
CKD	Baseline estimated glomerular filtration rate <60 ml/min/1.73 m^2^ (by MDRD formula) before admission

Abbreviations: DM, diabetes mellitus; CAD, coronary artery disease; HF, heart failure; PAOD, peripheral artery occlusive disease; PUD, peptic ulcer disease; COPD, chronic obstructive pulmonary disease; CKD, chronic kidney disease; MDRD, Modification of Diet in Renal Disease.

After enrollment, we dichotomized enrollees into those hospitalized ≤7 and >7 days (prolonged hospitalization), according to the literature [15]. All patients were followed until their discharge or death. Our primary endpoint was in-hospital mortality of these nonagenarians. A secondary endpoint was hospital length of stay, divided into ≤7 days and >7 days.

### Statistical Analysis

Statistical analysis was performed using SPSS 18.0 software (SPSS Inc., Chicago, IL). Categorical variables were expressed as numbers (percentages) and analyzed using the chi-square test, while continuous variables were expressed as the mean ± standard deviation, with comparisons between groups using the independent *t*-test. We subsequently utilized multivariate logistic regression analysis to investigate the independent predictors of in-hospital mortality with the stepwise selection method. All variables with a p value ≤0.1 in univariate analysis or variables that were deemed important were selected and entered into multivariate analysis. Another logistic regression analysis was utilized to determine the independent predictors of prolonged hospital stay (>7 days) from variables known at admission. The area under the receiver-operating curve was utilized in our modeling to ensure model quality. Kaplan-Meier survival curves were constructed according to factors identified in the logistic regression analysis. In all statistical analyses, a two-sided p<0.05 was considered statistically significant.

## Results

A total of 283 nonagenarians with 304 admissions were identified and recruited, among 3,600 patients admitted to AGMWs during this period. We only counted the first admission of each patient in the analysis. Their comorbidities at admission are displayed in Table 2. The average age of the enrollees was 92.9 years, with 46.3% male. Only one-fifth of nonagenarian in-patients had vascular comorbidities including DM, heart failure (HF) and old cerebrovascular accident (CVA), with even lower percentages of other co-existing illnesses.

**Table 2:** Clinical features of admitted nonagenarians by length of hospital stay (percentage in parentheses).

Variables*	Total (n = 283)	Hospital stay ≤7 days (n = 118)	Hospital stay >7 days (n = 165)	P value
Age (years)	92.9±3.2	92.7±3.1	93.0±3.3	0.406
Gender (% male)	131 (46.3)	60 (50.8)	71 (43.0)	0.195
*Comorbidities*				
Diabetes mellitus	54 (19.1)	18 (15.3)	36 (21.8)	0.167
CAD	26 (9.2)	11 (9.3)	15 (9.1)	0.947
Old AMI	3 (1.1)	2 (1.7)	1 (0.6)	0.380
HF	46 (16.3)	14 (11.9)	32 (19.4)	0.091
PAOD	10 (3.5)	5 (4.2)	5 (3)	0.589
Cirrhosis	3 (1.1)	2 (1.7)	1 (0.6)	0.380
PUD	20 (7.1)	7 (5.9)	13 (7.9)	0.530
COPD	36 (12.7)	17 (14.4)	19 (11.5)	0.473
Malignancy	26 (9.2)	12 (10.2)	14 (8.5)	0.630
Dementia	49 (17.3)	21 (17.8)	23 (17)	0.857
Old CVA	56 (19.8)	18 (15.3)	38 (23)	0.106
CKD	34 (12.0)	12 (10.2)	22 (13.3)	0.421
*Charlson Index (Age-adjusted)*	7.0±1.9	7.1±1.9	6.9±1.9	0.363
*Main diagnosis at admission*				0.109
Cardiovascular	30 (10.6)	11 (9.3)	19 (11.5)	
Pulmonology	116 (41.0)	55 (46.4)	61 (37.1)	
Gastroenterology	32 (11.3)	13 (11)	19 (11.5)	
Hepatobiliary	9 (3.2)	3 (2.7)	6 (3.6)	
Nephrology	69 (24.3)	25 (21.2)	44 (26.5)	
Infectious diseases	16 (5.7)	5 (4.2)	11 (6.8)	
Neurology illness	5 (1.8)	2 (1.7)	3 (1.9)	
Miscellaneous$	6 (2.1)	4 (3.5)	2 (1.1)	
*Concomitant illness at admission*				
Pneumonia	116 (41)	40 (33.9)	76 (46.1)	0.040
COPD with AE	12 (4.2)	7 (5.9)	5 (3)	0.234
Hypertension	8 (2.8)	3 (2.5)	5 (3)	0.808
HF with AE	21 (7.4)	7 (5.9)	14 (8.5)	0.421
Acute pancreatitis	1 (0.4)	0 (0)	1 (0.6)	0.399
UGI bleeding	24 (8.5)	11 (9.3)	13 (7.9)	0.669
LGI bleeding	1 (0.4)	0 (0)	1 (0.6)	0.399
Ileus	9 (3.2)	3 (2.5)	6 (3.6)	0.607
UTI	66 (23.3)	24 (20.3)	42 (25.5)	0.317
AKI	15 (5.3)	3 (2.5)	12 (7.3)	0.080
Ischemic stroke	5 (1.8)	2 (1.7)	3 (1.8)	0.938
Newly-found Malignancy	4 (1.4)	2 (1.7)	2 (1.2)	0.736
*Barthel Index at admission*	18.3±24.4	23.5±27.1	15.1±22.1	0.012
*Cardiopulmonary resuscitation*	1 (0.4)	1 (0.8)	0 (0)	0.238
*Do-Not-Resuscitate order*	103 (36.4)	41 (34.7)	62 (37.6)	0.627
*Hospital length of stay (days)*	11.4±10.1	4.7±1.8	16.2±10.8	<0.001

* All continuous variables were expressed in mean ± standard deviation, while dichotomized variables were expressed in frequency and percentages.

$ Including oncologic or endocrinologic illnesses.

Abbreviations: CAD, coronary artery disease; AMI, acute myocardial infarction; HF, heart failure; PAOD, peripheral artery occlusive disease; PUD, peptic ulcer disease; COPD, chronic obstructive pulmonary disease; CVA, cerebrovascular accident; CKD, chronic kidney disease; AE, acute exacerbation; UGI, upper gastrointestinal tract; LGI, lower gastrointestinal tract; UTI, urinary tract infection; AKI, acute kidney injury.

Main admission diagnoses were classified based upon category of systems rather than specific diseases, since elderly patients, especially those of extreme age, may have more vague symptomatology at initial presentation, making definite diagnosis at admission difficult. These extremely old patients might also be intolerant of or refuse examinations or invasive procedures during hospitalization, rendering definite diagnoses unachievable. Thus, since discharge diagnoses could suffer from a similar difficulty, we also listed main discharge diagnoses based upon category of systems. In classifying categories of systems, we first captured the organ-specific nature of such manifestations. If no organ-specific involvement was defined, we then proceeded to the second category of systems compatible.

The main admission diagnosis for enrollees was pulmonary diseases (41.0%), with nephrologic diseases ranking second (24.3%) (Table 2). Nonagenarians were least likely to be admitted with hepatobiliary (3.2%) or neurologic diseases (1.8%). Similarly, the most common concomitant diagnoses were pneumonia (41.0%), followed by UTI (23.3%), upper gastrointestinal tract (UGI) bleeding (8.5%) and HF (7.4%); other concomitant illnesses occurred in less than 5% of patients at admission. The admission Barthel Indexes were 18.3±24.4. After admission, few nonagenarian patients received CPR (0.4%), but more than one-third had a DNR code (36.4%).

The main discharge diagnoses were pulmonary diseases (45.0%) and nephrologic diseases (23.2%) (Table 3). The concomitant discharge diagnosis of pneumonia decreased (32.5%) from initial presentation, as did those of UTI (17.3%), UGI bleeding (8.5%) and ileus (2.1%). On the other hand, concomitant diagnoses of newly-found malignancy (3.5%) increased from initial presentation. The overall hospital mortality rate was 15.2% (43 patients).

**Table 3:** Discharge diagnoses of admitted nonagenarians by hospital length of stay (percentage in parentheses).

Variables*	Total (n = 283)	Hospital stay ≤7 days (n = 118)	Hospital stay >7 days (n = 165)	*p value*
*Main diagnosis at discharge*				0.116
Cardiovascular	22 (7.8)	7 (5.9)	15 (9.1)	
Pulmonology	127 (45)	59 (50.1)	68 (41.4)	
Gastroenterology	27 (9.5)	10 (8.5)	17 (10.3)	
Hepatobiliary	11 (3.9)	6 (5.1)	5 (3)	
Nephrology	66 (23.2)	23 (19.5)	43 (26.1)	
Infectious diseases	15 (5.3)	7 (5.9)	8 (4.8)	
Neurology illness	4 (1.4)	2 (1.7)	2 (1.2)	
Miscellaneous$	11 (3.9)	4 (3.7)	7 (4.2)	
*Concomitant diagnoses at discharge*				
Pneumonia	92 (32.5)	31 (26.3)	61 (37)	0.058
COPD with AE	11 (3.9)	5 (4.2)	6 (3.6)	0.797
Hypertension	4 (1.4)	1 (0.8)	3 (1.8)	0.497
HF with AE	21 (7.4)	7 (5.9)	14 (8.5)	0.421
Acute pancreatitis	3 (1.1)	1 (0.8)	2 (1.2)	0.769
UGI bleeding	18 (6.4)	7 (5.9)	11 (6.7)	0.804
LGI bleeding	2 (0.7)	1 (0.8)	1 (0.6)	0.812
Ileus	6 (2.1)	2 (1.7)	4 (2.4)	0.676
UTI	49 (17.3)	18 (15.3)	31 (18.8)	0.440
AKI	14 (4.9)	3 (2.5)	11 (6.7)	0.115
Ischemic stroke	5 (1.8)	1 (0.8)	4 (2.4)	0.323
Newly-found malignancy	10 (3.5)	4 (3.4)	6 (3.6)	0.912
*Barthel Index at discharge*	16.1±27.1	20.0±31.7	13.6±23.5	0.179

* All continuous variables were expressed in mean ± standard deviation, while dichotomized variables were expressed in frequency and percentages.

$ Including oncologic or endocrinologic illnesses.

Abbreviations: COPD, chronic obstructive pulmonary disease; AE, acute exacerbation; HF, heart failure; UGI, upper gastrointestinal tract; LGI, lower gastrointestinal tract; UTI, urinary tract infection; AKI, acute kidney injury.

Nonagenarians hospitalized for >7 days were similar to those hospitalized ≤7 days in age, gender and comorbidities (Table 2). Main admission diagnoses were also similar between patients with shorter and longer hospital stays (Table 2). However, patients with longer hospital stays had more concomitant pneumonia (p = 0.04. They also had a lower average Barthel Index (>7-day vs. ≤7-day, 15.1±22.1 vs. 23.5±27.1; p = 0.012). In terms of the hospital course, the CPR and DNR rates were similar for patients with shorter and longer hospital stays. The main discharge diagnoses were likewise similar between those with shorter and longer hospital stays. Patients with longer hospital stays were slightly more likely to have concomitant pneumonia as a discharge diagnosis (p = 0.058), but no difference existed in other concomitant discharge diagnoses. The functional status of patients by length of stay also did not differ at discharge (p = 0.179) (Table 3).

**Figure 1:**
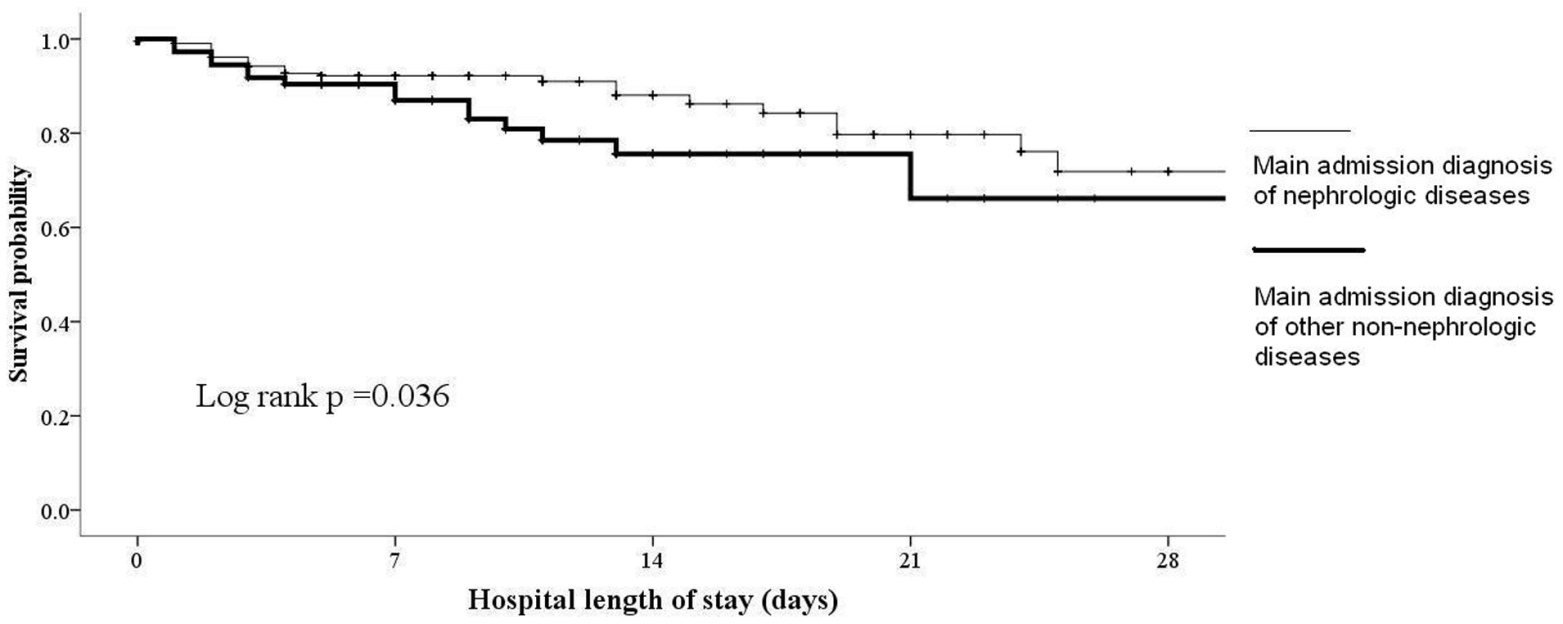
Kaplan-Meier survival curve based on category of main admission diagnosis (nephrologic vs. non-nephrologic diseases). Nephrologic diseases included the diagnosis or management of diseases comprised of acute/chronic renal dysfunction, urinary tract infection (upper/lower), and dyselectrolytemia.

**Figure 2:**
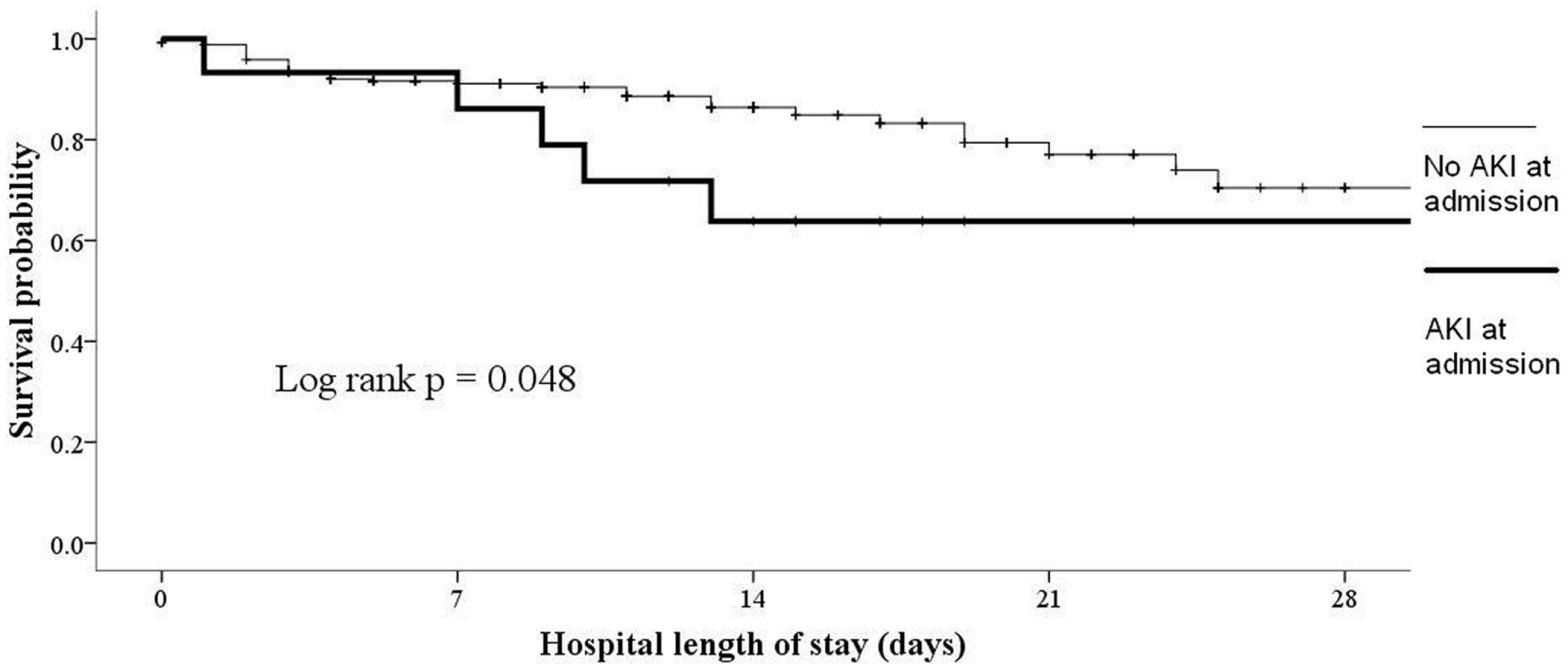
Kaplan-Meier survival curve based upon presence of AKI or not at admission. Abbreviation: AKI, acute kidney injury.

Univariate analysis showed that survivors were significantly older than non-survivors (p = 0.046) and had higher Barthel Index at admission (p<0.001) and discharge (p = 0.006) (Table 4). Non-survivors had significantly more AKI at admission (p = 0.044) and discharge (p = 0.028). Multivariate logistic regression analysis was subsequently performed to identify the independent predictors for in-hospital mortality in nonagenarian in-patients (Table 5). Variables included in the regression model consisted of age, gender, comorbidities, main diagnostic categories at admission/discharge, concomitant illnesses at admission/discharge, Barthel Index at admission/discharge and receipt of CPR. After adjustment, lower Barthel Index at admission, a main admission diagnosis of nephrologic diseases and AKI at admission were associated with higher hospital mortality (for Barthel Index, odds ratio [OR] 0.93 per score, 95% confidence interval [CI] 0.89–0.98, p = 0.005; for main diagnosis of nephrologic diseases, OR 4.83, 95%CI 1.33–17.47, p = 0.016; for AKI, OR 30.7, 95%CI 2.59–360.7, p = 0.007). Another logistic regression analysis to determine factors linked to hospital stays >7 days was performed, taking into account age, gender, comorbidities, main diagnostic categories, concomitant illnesses at admission, admission Barthel Index, receipt of CPR and DNR order. Comorbid HF (OR 3.05, 95%CI 1.02–9.15, p = 0.046) and low admission Barthel Index (OR 0.98 per score, 95%CI 0.97–0.99, p = 0.048) were associated with longer hospitalization. We constructed Kaplan-Meier survival curves for in-hospital mortality based on factors identified in Table 5 (Figure 1 and 2). These curves showed that the presence of nephrologic diseases or AKI at admission was associated with increased risk for in-hospital mortality.

**Table 4:** Univariate analysis of factors associated with nonagenarian in-patient hospitalization mortality (percentage in parentheses).

Variables*	Total (n = 283)	Survivors (n = 240)	Non-survivors (n = 43)	*p value*	
*Age (year)*	92.9±3.2	92.7±3.1	93.8±3.9	0.046	
*Gender (male%)*	131 (46.3)	115 (47.9)	16 (37.2)	0.196	
*Comorbidities*					
Diabetes mellitus	54 (19.1)	47 (19.6)	7 (16.3)		0.613
CAD	26 (9.2)	22 (9.2)	4 (9.3)		0.977
Old AMI	3 (1.1)	2 (0.8)	1 (2.3)		0.381
HF	46 (16.3)	38 (15.8)	8 (18.6)		0.652
PAOD	10 (3.5)	9 (3.8)	1 (2.3)		0.643
Cirrhosis	3 (1.1)	3 (1.3)	0 (0)		0.463
PUD	20 (7.1)	17 (7.1)	3 (7)		0.980
COPD	36 (12.7)	34 (14.2)	2 (4.7)		0.085
Malignancy	26 (9.2)	21 (8.8)	5 (11.6)		0.549
Dementia	49 (17.3)	42 (17.5)	7 (16.3)		0.846
Old CVA	56 (19.8)	51 (21.3)	5 (11.6)		0.146
CKD	34 (12)	31 (12.9)	3 (7)		0.272
*Main diagnosis at admission*					
Cardiovascular	30 (10.6)	25 (10.4)	5 (11.6)		0.813
Pulmonology	116 (41)	100 (41.7)	16 (37.2)		0.586
Gastroenterology	32 (11.3)	28 (11.7)	4 (9.3)		0.653
Hepatobiliary	9 (3.2)	8 (3.3)	1 (2.3)		0.730
Nephrology	69 (24.4)	56 (23.3)	13 (29.9)		0.025
Infectious diseases	16 (5.7)	13 (5.4)	3 (7)		0.685
Neurology illness	5 (1.8)	4 (1.7)	1 (2.3)		0.764
Miscellaneous$	6 (2.1)	6 (2.5)	0 (0)		0.125
*Concomitant illness at admission*					
Pneumonia	116 (41)	99 (41.3)	17 (39.5)	0.834	
COPD with AE	12 (4.2)	11 (4.6)	1 (2.3)	0.500	
Hypertension	8 (2.8)	7 (2.9)	1 (2.3)	0.830	
HF with AE	21 (7.4)	17 (7.1)	4 (9.3)	0.611	
Acute pancreatitis	1 (0.4)	1 (0.4)	0 (0)	0.673	
UGI bleeding	24 (8.5)	20 (8.3)	4 (9.3)	0.834	
LGI bleeding	1 (0.4)	1 (0.4)	0 (0)	0.673	
Ileus	9 (3.2)	8 (3.3)	1 (2.3)	0.730	
UTI	66 (23.3)	50 (20.8)	16 (37.2)	0.019	
AKI	15 (5.3)	10 (4.2)	5 (11.6)	0.044	
Ischemic stroke	5 (1.8)	4 (1.7)	1 (2.3)	0.764	
Newly-found malignancy	4 (1.4)	3 (1.3)	1 (2.3)	0.584	
*Main diagnosis at discharge*					
Cardiovascular	22 (7.8)	19 (7.9)	3 (7)	0.833	
Pulmonology	127 (45)	107 (44.6)	20 (47.2)	0.741	
Gastroenterology	27 (9.5)	25 (10.4)	2 (4.7)	0.237	
Hepatobiliary	11 (3.9)	10 (4.2)	1 (2.3)	0.567	
Nephrology	66 (23.2)	57 (23.8)	9 (20.4)	0.611	
Infectious diseases	15 (5.3)	11 (4.6)	4 (9.3)	0.205	
Neurology illness	4 (1.4)	4 (1.7)	0 (0)	0.396	
Miscellaneous$	11 (3.9)	7 (2.9)	4 (9.3)	0.062	
*Concomitant diagnoses at discharge*					
Pneumonia	92 (32.5)	75 (31.3)	17 (39.5)	0.287	
COPD with AE	11 (3.9)	11 (4.6)	0 (0)	0.153	
Hypertension	4 (1.4)	3 (1.3)	1 (2.3)	0.584	
HF with AE	21 (7.4)	19 (7.9)	2 (4.7)	0.454	
Acute pancreatitis	3 (1.1)	2 (0.8)	1 (2.3)	0.381	
UGI bleeding	18 (6.4)	16 (6.7)	2 (4.7)	0.619	
LGI bleeding	2 (0.7)	2 (0.8)	0 (0)	0.550	
Ileus	6 (2.1)	6 (2.5)	0 (0)	0.296	
UTI	49 (17.3)	46 (19.2)	3 (7)	0.052	
AKI	14 (4.9)	9 (3.8)	5 (11.6)	0.028	
Ischemic stroke	5 (1.8)	5 (2.1)	0 (0)	0.341	
Newly-found malignancy	10 (3.5)	7 (2.9)	3 (7)	0.185	
*Barthel index at admission*	18.3±24.4	20.7±25.4	4.7±9.3	<0.001	
*Barthel index at discharge*	16.1±27.1	18.6±28.3	2.1±4.3	0.006	
*Cardiopulmonary resuscitation*	1 (0.4)	0 (0)	1 (2.3)	0.018	
*Do-Not-Resuscitation order*	103 (36.4)	68 (28.3)	35 (81.4)	<0.001	

* All continuous variables were expressed in mean ± standard deviation, while dichotomized variables were expressed in frequency and percentages.

$ Including oncologic or endocrinologic illnesses.

Abbreviations: CAD, coronary artery disease; AMI, acute myocardial infarction; HF, heart failure; PAOD, peripheral artery occlusive disease; PUD, peptic ulcer disease; COPD, chronic obstructive pulmonary disease; CVA, cerebrovascular accident; CKD, chronic kidney disease; AE, acute exacerbation; UGI, upper gastrointestinal tract; LGI, lower gastrointestinal tract; UTI, urinary tract infection; AKI, acute kidney injury.

**Table 5:** Multivariate logistic regression analysis of the predictors of hospitalization duration and in-hospital mortality in nonagenarians, with stepwise selection of variables (n = 283).

Variables*	OddsRatio	95% ConfidenceInterval	*p* Value	Variables#	Odds Ratio	95% Confidence Interval	*p* value
**Heart failure**	3.05	1.02–9.15	0.046				
**Barthel Index (admission)** **(per one score)**	0.98	0.97–0.99	0.021	**Barthel Index (admission)** **(per one score)**	0.93	0.89–0.98	0.005
				**Nephrology admission**	4.83	1.33–17.47	0.016
				**Acute kidney** **injury (admission)**	30.7	2.59–360.7	0.007

* Variables included in the analysis were age, gender, comorbidities (DM, CKD, old CVA, dementia, heart failure, COPD, malignancy), Barthel Index (at admission), main diagnosis at admission and concomitant diagnosis at admission and receipt of CPR; estimated Area under receiver operating curve (AUROC) = 0.787.

# Variables included in the analysis comprised of age, gender, comorbidities (DM, CKD, old CVA, dementia, heart failure, COPD, malignancy), Barthel Index (at admission and discharge), main diagnosis at admission and discharge, concomitant illnesses at admission and discharge and receipt of CPR; estimated AUROC = 0.752.

Abbreviations: DM, diabetes mellitus; CKD, chronic kidney disease; CVA, cerebrovascular accident; COPD, chronic obstructive pulmonary disease; CPR, cardiopulmonary resuscitation.

## Discussion

In the current study, we utilized a cohort of nonagenarian in-patients to investigate the determinants of hospitalization length of stay and mortality in these understudied patients. We found that lower admission functional status was associated with longer hospital stays and higher hospital mortality. Main admission diagnosis of nephrologic diseases or AKI was associated with poor hospitalization outcome in nonagenarians, while baseline heart failure signified the possibility of prolonged admission.

Many clinicians are already aware of the clinical difference between the old and the young, including physiologic organ degeneration, emergence of comorbidities, the detrimental effect on medical interventions, and the functional decline associated with aging [16]. However, even within the old, it would be erroneous to extrapolate findings from the younger-old to the oldest-old [7], [16]. For example, nonagenarians maintain better social relationships with their families and have higher levels of life satisfaction than septuagenarians, both parameters related to patient outcomes [17], [18]. Researchers have classified surviving centenarians into three types according to different phenotypes: “survivors,” those with diagnoses of age-associated diseases (including heart diseases, stroke or skin cancer) before the age of 80; “delayers,” those who acquire their diagnoses of age-associated diseases at or after the age of 80, far beyond their concurrent birth cohort’s life expectancy; and “escapers,” those remain alive over 100 years without the diagnosis of age-associated diseases [19], [20]. Consequently, in considering this “compression of morbidity” theory, evaluation of functional status, in addition to comorbidities, is necessary for determining the outcome of patients who survive to extreme ages [21], [22]. Our nonagenarian cohort has a relatively low frequency of comorbidities at baseline, bearing features akin to the “escapers” group [20]. This group has minimal age-related comorbidities (hypertension, DM, stroke, chronic obstructive pulmonary disease [COPD], cancers) even past their 80–90 s, and this longevity phenotype may explain the difference between our patients and younger ones: our nonagenarians may demonstrate higher resistance during medical illnesses [20].

Our cohort of nonagenarians had a similar in-hospital mortality rate (15.2%) to those of previous reports (17–22%), but different mortality predictors [23]–[25]. Past research has mostly focused on long-term outcomes in community-dwelling nonagenarians, and few have investigated short-term goals (e.g., discharge from acute care hospitals) [4]. Likewise, determinants of outcomes for nonagenarians may also differ between community residents and hospitalized patients. Nybo et al. found that cardiovascular disease and DM predict mortality in community-dwelling nonagenarians, but not marital status, smoking/alcohol consumption and obesity; Conde-Martel et al., using a cohort of hospitalized nonagenarians, found that Barthel Index and heart failure were the only determinants of mortality after discharge [7], [8]. Several studies have also found that acute coronary syndrome at presentation was not associated with worse survival, but sphincter incontinence and being functionally dependent pre-admission were more important [23], [24]. It is then clear that different factors can determine in-hospital and long-term mortality among nonagenarians in different settings. Our results may help to uncover additional risk factors important for in-patient mortality among nonagenarians and centenarians.

In this study, we discovered that lower functional status – independent of comorbidities, admission diseases or complications – is associated with longer hospital stays and higher hospital mortality. Functional status, or the level of disability, has been repeatedly shown to predict both short-term and long-term mortality in nonagenarians [7], [8], [23]. Functional status is reportedly a surrogate for outcome-affecting comorbidities, and pre-admission low functional status could predispose surgery patients to further decline after discharge [26], [27]. Furthermore, functional status could also reflect the interplay/interaction of various illnesses and the relationship with potential psychosocial factors, which indirectly modify clinical outcomes [28], [29]. However, the impact of the Barthel Index score on the elderly seems to differ between the general population of elderly in-patients and nonagenarians. The mortality risk is higher for patients with higher Barthel Index (I.e., the less disabled), but lower for those with lower scores [8], [30], [31]. We believe this phenomenon is unique and deserves further investigation, suggesting as it does that the influence of most outcome-predictive factors may be diminished in the extremely old.

In addition to its influence on hospital mortality, we found that lower functional status is also associated with prolonged hospital stays (those >7 days). This issue has been scarcely addressed before in the elderly population, and not at all in nonagenarians. Gambier et al. found that elderly patients with lower ADL scores often have longer hospital stays [32]. Another systemic review also disclosed that, in the elderly, more functional disability and longer hospital stays are significantly associated with subsequent hospital re-admissions, adverse events and poorer outcomes [33]. In our study, poorer ADL status may indirectly lead to worsening outcomes in nonagenarians, since patients with lower functional status also have a higher likelihood of pneumonia at admission, resulting in the need for specialized care and complicated disease management (Table 2). This unrelenting course culminates in prolonged hospital stays and higher mortality [34]. As our results suggest, such phenomena may be particularly pronounced in this extremely old population [35], [36].

The intriguing finding that comorbid heart failure is associated with longer hospital stays in nonagenarians has not been reported before. Factors such as activity levels, severity of comorbidities, nutritional status, and amount of lean body mass have been demonstrated to predict hospitalization length of stay in geriatric patients [37]. Elderly heart failure patients, through the interplay of reduced physical activity and the endocrine milieu of inappropriate neurohormonal activation, are at risk for sarcopenia, malnutrition and deterioration of physical function [38], [39]. All of the above factors can potentially result in prolonged hospital stays. In addition, the complex medication regimens of heart failure patients further increase the risk of prolonged hospitalization in such patients [40]. Social factors may also play a role in this process [41].

Admission with a main diagnosis of nephrologic diseases and AKI at presentation was found to be an important predictor of mortality in our nonagenarians. Indeed, admission diagnostic group is an essential aspect of devising proper treatment in the geriatric population [42]. Main admission diagnoses of nephrologic diseases in geriatrics consists of the diagnosis and management of acute/chronic renal dysfunction, UTI (upper/lower), and dyselectrolytemia. Each of these components helps determine the hospitalization outcomes in the old: dysnatremia and dyskalemia at elderly admission is associated with in-hospital mortality [43], [44], while UTI contributes to higher morbidity and mortality in the institutionalized old [45]. Furthermore, older patients have more limited renal reserve and are prone to develop AKI [46], [47]. AKI impairs the survival of elderly in-patients by deranging the body fluid/electrolyte balance, and this impairment is expectedly exaggerated in the extremely old [48]–[50]. Consequently, nonagenarians with a main admission diagnosis of nephrologic diseases and AKI are at risk for adverse outcomes compared with other patients of similar age. They must be managed carefully, in light of the significant adverse impact of renal dysfunction on overall health.

### Limitations

Our study has its strength. The issue of hospital length of stay in nonagenarians has not been specifically dealt with in the literature, and our finding is among the first in this field. However, there are also limitations. First, the single-center nature of the study partially limits the generalizability of our findings. However, this defect is partially negated by the fact that the range of patients we admitted came from all over the country, making them thus representative of the nonagenarian Asian population. Second, the main diagnoses at admission and discharge were decided by the attending physicians. However, we have pre-trained all staff during ward care, and inter-rater variability was found to be very low (<5%). These diagnoses were verified and confirmed for consistency by two independent case managers.

### Conclusion

From a cohort of hospitalized nonagenarians, we identified potential determinants of prolonged hospital stay and in-hospital mortality. Baseline comorbid heart failure may be associated with hospitalization longer than 7 days, while nephrologic diseases as the main admission diagnoses and AKI at presentation correlate with increased in-hospital mortality. Further studies are needed to confirm our results and also assist in designing appropriate strategies for the clinical management of this ever-growing population.
